# Aerobic Degradation of Trichloroethylene by Co-Metabolism Using Phenol and Gasoline as Growth Substrates

**DOI:** 10.3390/ijms15059134

**Published:** 2014-05-22

**Authors:** Yan Li, Bing Li, Cui-Ping Wang, Jun-Zhao Fan, Hong-Wen Sun

**Affiliations:** MOE Key Laboratory of Pollution Processes and Environmental Criteria, College of Environmental Science and Engineering, Nankai University, Tianjin 300071, China; E-Mails: liyanid@hotmail.com (Y.L.); libingnankai@163.com (B.L.); wangcp@nankai.edu.cn (C.-P.W.); cookcoolcc@126.com (J.-Z.F.)

**Keywords:** trichloroethylene, co-metabolism, phenol, combined pollution, gasoline

## Abstract

Trichloroethylene (TCE) is a common groundwater contaminant of toxic and carcinogenic concern. Aerobic co-metabolic processes are the predominant pathways for TCE complete degradation. In this study, *Pseudomonas fluorescens* was studied as the active microorganism to degrade TCE under aerobic condition by co-metabolic degradation using phenol and gasoline as growth substrates. Operating conditions influencing TCE degradation efficiency were optimized. TCE co-metabolic degradation rate reached the maximum of 80% under the optimized conditions of degradation time of 3 days, initial OD_600_ of microorganism culture of 0.14 (1.26 × 10^7^ cell/mL), initial phenol concentration of 100 mg/L, initial TCE concentration of 0.1 mg/L, pH of 6.0, and salinity of 0.1%. The modified transformation capacity and transformation yield were 20 μg (TCE)/mg (biomass) and 5.1 μg (TCE)/mg (phenol), respectively. Addition of nutrient broth promoted TCE degradation with phenol as growth substrate. It was revealed that catechol 1,2-dioxygenase played an important role in TCE co-metabolism. The dechlorination of TCE was complete, and less chlorinated products were not detected at the end of the experiment. TCE could also be co-metabolized in the presence of gasoline; however, the degradation rate was not high (28%). When phenol was introduced into the system of TCE and gasoline, TCE and gasoline could be removed at substantial rates (up to 59% and 69%, respectively). This study provides a promising approach for the removal of combined pollution of TCE and gasoline.

## 1. Introduction

Trichloroethylene (TCE) has been widely used as a solvent for the past 70 years. Because of improper disposal practices for spent solvents in the past, TCE has become a major contaminant in groundwater [1]. Notably, an estimated 852 out of 1430 National Priorities List sites identified by the US EPA are contaminated with TCE [2]. In China, the annual production of TCE was 2.5 × 10^5^ ton in 2011, and this was estimated to increase by 40% in 2013 [3]. TCE is listed as one of the 68 priority pollutants in aquatic environment. TCE pollution in groundwater has been reported in several cities, such as Kaifeng, Henan province; Xuzhou, Jiangsu province; and Shanghai [4–6]. TCE concentration up to 63.7 μg/L has been reported in Beijing [7].

Due to the established toxicity of this product, the US EPA proposed a non-enforceable grant level goal of 0 μg/L for TCE in drinking water. Presently, the enforced maximum level for TCE is 5 μg/L [8]. In China, TCE belongs to non-compulsive indices in the standard for drinking water, and its limiting value is 0.07 mg/L. Therefore, attempts have been made to find practical approaches to remove TCE. Reductive dechlorination under anaerobic conditions and aerobic co-metabolic processes are the predominant pathways for TCE biotransformation [9]. The anaerobic degradation is not complete, and possible accumulation of known carcinogen of vinyl chloride (VC) is a main limitation on its application. Hence, there has been much interest in developing aerobic *in-situ* biotransformation processes for TCE degradation since Wilson and Wilson first demonstrated the co-metabolism of TCE in soil columns [10,11]. In co-metabolism, a type of key enzymes, oxygenases, used by the microorganisms to initiate the oxidation of primary substrates, are generated by the stimulation of primary substrates, and can fortuitously transform TCE and many other chlorinated aliphatic hydrocarbons. Initially, methane was used as a primary substrate for the aerobic co-metabolism of TCE and its less chlorinated metabolites [12,13]; however the removal rate was rather low for TCE. Therefore, other potential growth substrates were sought, and phenol was found to be quite superior to methane for TCE degradation [14,15].

In the real environment, sites are often polluted by multiple pollutants, called combined pollution. In order to deal with the possible combined pollution, Balasubramanian *et al.* [16] recently checked the co-metabolic degradation of chloroform in binary, ternary and quaternary systems containing some non-chlorinated solvents commonly used in pharmaceutical industries, such as small alcohols, acetone, acetonitrile, and toluene, and they found the co-metabolic degradation of chloroform occurred in the presence of acetone and toluene. Another study checked the effect of chlorinated solvents on the microbial degradation of dioxane (a stabilizer for chlorinated solvents) and the simultaneous co-metabolism of the chlorinated solvents because of their co-occurrence in groundwater [17]. The advantage of using co-occurring or common pollutants as growth substrates for TCE co-metabolism is the avoidance or reduction of the introduction of new pollutants into the environment, and the possibility for several kinds of pollutants to be removed simultaneously. Gasoline is a common kind of pollutant in groundwater and there is a possibility that a site is co-polluted by gasoline and TCE, or that the sites polluted by TCE and gasoline might be very close to each other. Gasoline is a mixture of aliphatic paraffins, cycloparaffins, mono-olefins, aromatics, cyclo-olefins, diolefins, and acetylenes [18]. The multiple, easily oxidizable substrates in this mixture endow its potential to be a candidate growth substrate for TCE co-metabolism. Related studies can illustrate whether TCE and gasoline can be removed simultaneously; however, these kinds of studies are rather scarce [19].

The objective of this study was to investigate the potential of phenol and gasoline as growth substrates for TCE co-metabolism using *Pseudomonas fluorescens* as an active species. The influence of several operating parameters on TCE co-metabolic degradation was studied systematically with the effect of the co-substrate being studied for the first time. TCE transformation capacity and transformation yield were calculated to evaluate the efficiency of TCE co-metabolism, which makes it easier to compare with other studies. Activities of several enzymes and possible intermediates were measured to illustrate the possible co-metabolism mechanism. Finally, to improve the co-removal of combined pollution of TCE and gasoline, co-metabolism in ternary and quaternary systems with phenol and nutrient broth as additional growth substrates were checked.

## 2. Results and Discussion

Systematic experiments were conducted to study the co-metabolic degradation of TCE by *P. fluorescens* using phenol and gasoline as growth substrates. First, operating conditions (including degradation time, cell density, phenol concentration, TCE initial concentration, pH, salinity and co-substrate) influencing TCE co-metabolic degradation rate using phenol as growth substrate were optimized. TCE transformation efficiencies were evaluated by changing a specific condition parameter and setting other parameters at fixed values. After all parameters were optimized, the experiments were repeated by changing one specific parameter within the designed range and setting other parameters at their optimized values. Then, to illustrate the possible mechanisms, kinetic changes of potential key enzymes were determined and less chlorinated products and chlorine ions were also measured. Finally, binary, ternary and quaternary co-metabolic degradation systems of phenol, TCE, nutrient broth, and gasoline were studied to set up a technique to remove co-pollution of TCE and gasoline.

### 2.1. Influence of Operating Conditions on TCE Co-Metabolic Degradation Efficiency

**Figure 1.**
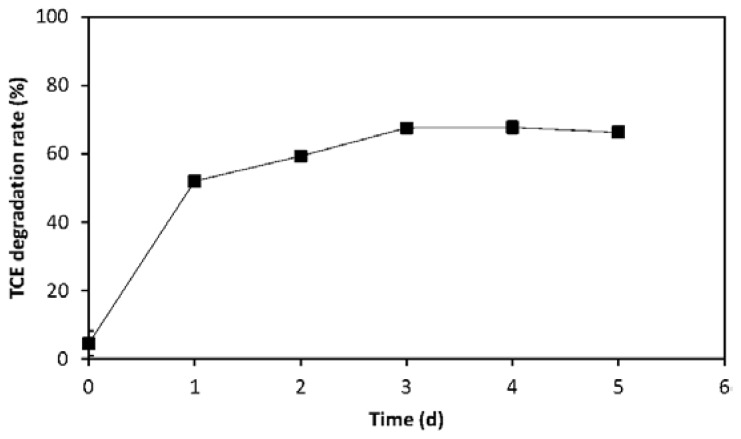
The kinetics of co-metabolic degradation of 1 mg/L TCE by *P. fluorescens* in the presence of 100 mg/L phenol.

To find the time to reach steady state, the kinetics of TCE co-metabolic degradation were measured in the presence of 100 mg/L phenol for 5 days (Figure 1). It was found that TCE declined quickly in the initial three days, and then it did not decrease obviously. Hence, a 3-day test period was selected in the following condition optimizing experiments.

**Figure 2.**
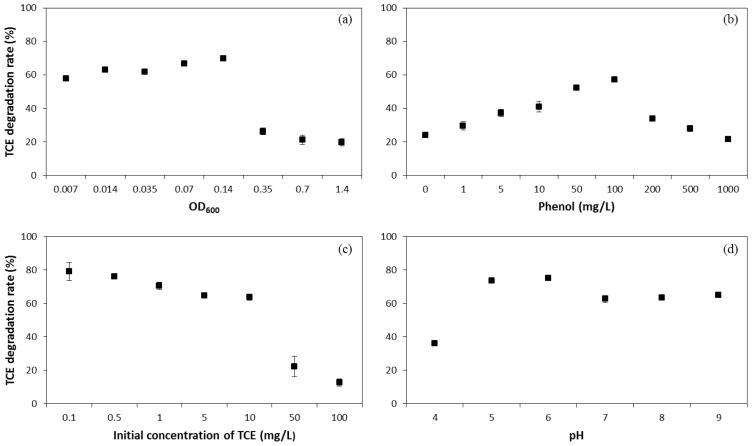
Influences of cell initial density (**a**); phenol concentration (**b**); initial concentration of TCE (**c**) and pH (**d**) on TCE co-metabolic degradation rate by *P. fluorescens* in the presence of phenol.

Effects of cell initial density, phenol concentration, initial concentration of TCE, and pH on TCE co-metabolic degradation efficiency by *P. fluorescens* in the presence of phenol were studied (Figure 2). The TCE degradation rate increased slightly from 58% to 70% when the initial OD_600_ of the culture increased from 0.007 to 0.14, which represents 6.3 × 10^5^–1.26 × 10^7^ cell/mL microorganism density (Figure 2a). Further increasing cell initial density to 1.4 (1.26 × 10^8^ cell/mL) led to a reduction in TCE degradation. This is because the greater initial cell density made the oxygen supply more quickly, exhausted, leading to a reduction in microorganism activity, and even death. At the end of the experiment, the oxygen in these systems ranged from 0.53 to 3.63 mg/L, decreasing with increasing initial microorganism density. To avoid the loss of TCE by evaporation, the experiment in this study was designed as a closed system. In real remediation projects in field, oxygen was provided to accelerate the aerobic metabolism, which is called bioventing. It was reported that the number of bacteria increased substantially during co-metabolic bioventing [20]; along with the enhancement in biodegradation, evaporation into ambient air will increase simultaneously. The changes in the two processes are not linear, and hence it is possible to select an optimized oxygen injection rate to achieve the maximum biodegradation rate at an acceptable evaporation rate.

Initial concentration of phenol was adjusted from 0 to 1000 mg/L to check its effect on TCE degradation (Figure 2b). The addition of phenol promoted TCE degradation, and the degradation rate increased from 24% to 57% when phenol concentration increased from 0 to 100 mg/L. This proved the involvement of a co-metabolism mechanism. Further increase in phenol concentration led to a reduction in TCE degradation. As for the reason for this reduction, the possible toxicity incurred by high phenol concentration was excluded since the OD_600_ did not decline [16]. Further, the oxygen was not depleted, and an oxygen concentration of 3.96 mg/L was measured. The probable reason may be that phenol inhibited TCE co-metabolic degradation through occupying active sites of oxidizing enzymes competitively. Co-metabolism is a complex phenomenon, where the co-existence of growth substrates is a premise of the degradation of non-growth substrates by stimulating active enzymes. However, the growth substrate may act as a competitor for the non-growth substrate for the active sites of the generated enzyme beyond a specific threshold value [21,22]. A similar observation was reported for toluene and TCE by Landa *et al.* [23]. Hence, the relative concentrations of the growth and non-growth substrates are crucial, and an optimum ratio exists.

The initial concentration of TCE was adjusted from 0.1 to 100 mg/L and the phenol concentration was fixed at 100 mg/L, to check the influence of TCE initial concentration on its degradation rate (Figure 2c). TCE co-metabolic degradation rate decreased with increasing TCE initial concentration. Satisfying results were obtained when TCE initial concentration was within 0.1–10 mg/L with the degradation rate between 79% and 64%. However, the degradation rate was unsatisfactory when TCE initial concentration exceeded 50 mg/L. Chen *et al.* [24] implied that a self-inhibition of TCE is present in the system at higher TCE concentrations (>6 mg/L), and high concentration of TCE may have influence on the affinity of substrate and enzyme.

The pH of mineral salt medium was adjusted in the range of 4–9 to find the optimum acid-base condition (Figure 2d). TCE degradation rate reached the maximum (73%–75%) under faintly acidic conditions (pH = 5–6). TCE degradation rate results (63%–65%) could be obtained under the neutral or alkalescent condition (pH = 7–9). TCE degradation rate was the lowest (36%) at pH 4. This may be due to the lowest survival rate of *P. fluorescens* at this extreme pH, and a poor viability state was observed under the microscope. Liu *et al.* [25] reported that the optimal living condition of *P. fluorescens* was neutral and weakly basic environments. From the point of enzyme activity, catechol 1,2-dioxygenase was the key enzyme involved in TCE co-metabolism (Section 2.3). The pH was found to influence the activity of catechol 1,2-dioxygenase. Brivio *et al.* [26] found that catechol 1,2-dioxygenase activity is independent of pH, in the pH range of 5.2–8.6. Silva *et al.* [27] reported that catechol 1,2-dioxygenase activity was very low when pH value equaled to 4. These results may explain why the TCE degradation rate was satisfying when pH value changed from 5 to 9 and was not when the pH value equaled 4. The coastal area in eastern China is the most advanced economic area, and the most severely contaminated. The soil and groundwater in large parts of northeastern China are saline-alkali. The results described here provide a promising approach for TCE remediation in this area.

Moreover, the effect of the salinity of mineral salt medium (0.045%–3%) on TCE co-metabolic degradation was also checked. Generally, the change in TCE degradation rate was not high within the tested salinity range (data not shown). Satisfying TCE degradation rates of 63% to 75% could be obtained in the tested salinity range with the maximum of 78% at salinity of 0.1%. This endows the applicability of this method in the remediation of high-saline areas, *i.e.*, seashore areas.

**Figure 3.**
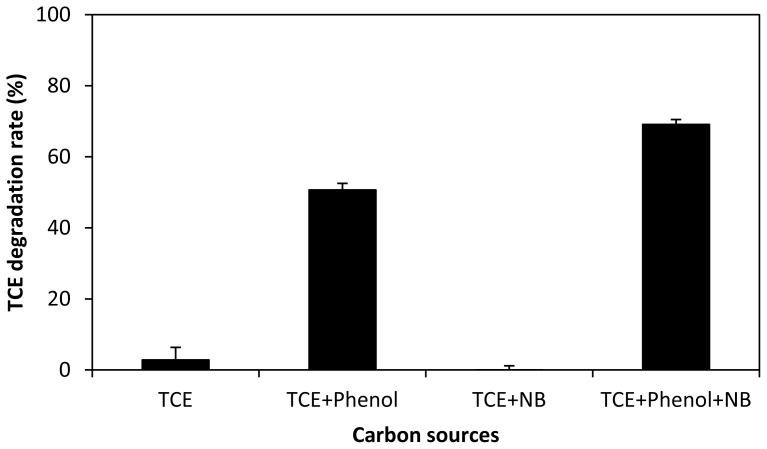
Influence of different carbon sources as growth substrates on TCE co-metabolic degradation by *P. fluorescens*.

Finally, the effect of nutrient broth (NB) on TCE co-metabolic degradation was checked to evaluate the potential of different carbon sources as growth substrate (Figure 3). TCE was not degraded when NB was added as the sole growth substrate but was degraded when phenol was the sole carbon and energy source. This suggests that phenol stimulated *P. fluores-cens* to generate the crucial enzyme for TCE metabolism. Simultaneous addition of NB and phenol improved TCE co-metabolic degradation efficiency further as compared to phenol alone. This suggests that NB could enhance the growth of the microorganisms but cannot stimulate the generation of the key enzyme. A similar result was observed by Wang and Loh [28], who found that although sodium glutamate (SG), a common growth medium cannot act as the growth source for metabolic degradation of 4-chlorophenol, the addition of SG can minimize the requirement for the growth substrate, phenol. This suggests that the interaction of substrates involved in co-metabolism is quite complicated, which can be managed during *in-situ* remediation. In fact, most of TCE-co-metabolism active microorganisms are capable of growing on many substrates. However, only some of the substrates like phenol may stimulate the expression of the TCE-degrading oxygenase, whereas others like NB can-not [29].

### 2.2. Evaluation of TCE Transformation Efficiency

Based on the mass of TCE transformed, the cumulative mass of growth substrate consumed, and the increase in cell mass, the modified transformation capacity (*T*′_c_) and transformation yield (*T*′_y_) were calculated [30]:

$$\begin{array}{c} {T^{\prime }}_{\text{c}}={X^{\prime }}_{\text{c}}/{X^{\prime }}_{\text{m}}\\ {T^{\prime }}_{\text{y}}={X^{\prime }}_{\text{c}}/S \end{array}$$

where *X*′_m_ is the increase in biomass during the incubation period (mg/L); *S* is the cumulative amount of the primary substrate (phenol in this study) consumed (mg/L); and *X*′_c_ is the cumulative amount of the target compound (TCE in this study) degraded (μg/L) with the calibration by the control.

In the system of phenol as growth substrate, *T*′_c_ equaled to 20 μg/mg. In the system of phenol and NB as growing substrates together, *T*′_c_ equaled 1.6 μg/mg. It can be seen that the addition of NB promoted TCE co-metabolism rate by 18%, but exhibited the transformation capacity per unit mass of cells. The addition of NB played a significant role in proliferating cells, but made less of a contribution to improving TCE degradation efficiency. Large amounts of proliferated cells could not co-metabolize TCE, only those using phenol as growth substrate can generate the key enzyme to degrade TCE. Therefore, the primary reason of the NB promotion effect on TCE co-metabolic degradation is to increase the cells which generated active enzyme stimulated by phenol.

In the system of phenol as growth substrate, *T*′_y_ equaled to 5.1 μg/mg. In the system of phenol and NB as growth substrates simultaneously, *T*′_y_ equaled to 6.9 μg/mg. Phenol was consumed completely in both systems. The addition of NB improved TCE co-metabolic transformation yield (*T*′_y_). Elango *et al.* [30] studied the co-metabolism of TCE using several monoaromatic compounds, benzene, chlorobenzene (CB), and dichlorobenzene isomers (DCBs), as growth substrates. Isolates that grew on benzene, CB, 1,2-DCB and 1,3-DCB were identified as *Rhodococcus*, *Ralstonia*, *Variovorax* and *Ralstonia* spp., respectively. *T*′_c_ and *T*′_y_ were calculated to be 0.15–0.33 and 0.06–0.11 μg/mg, respectively. These values are lower than those acquired in this study, suggesting that phenol together with *P. fluorescens* is more efficient than the above systems.

### 2.3. Key Enzymes and Products in TCE Co-Metabolic Degradation System

**Figure 4.**
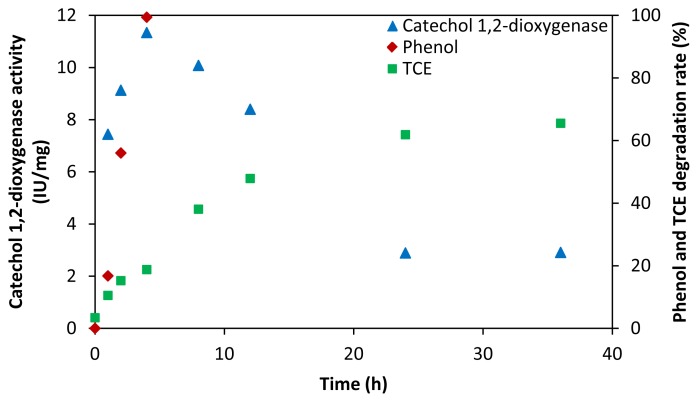
Catechol 1,2-dioxygenase activity and degradation efficiencies of TCE and phenol at different time during TCE degradation by *P. fluorescens* in the presence of 100 mg/L phenol.

It has been well recognized that during co-metabolism, the active microorganism was stimulated by the growth substrate to generate key enzymes, which are capable of degrading the target compound [31,32]. In the co-metabolism of TCE, alkene monooxygenase [33], phenol hydroxylase [34], particulate methane monooxygenase [35], soluble methane monooxygenase [36], toluene dioxygenase [37] and toluene monooxygenase [38] have been reported to be the active enzymes. The catechol dioxygenases catalyze the oxidative cleavage of catechol and substituted catechols, a key step in the bacterial degradation of aromatic compounds in the environment [39,40]. Two families of dioxygenase enzyme were discovered by Hayaishi *et al.* [41,42], which can catalyze the oxidative cleavage of catechol, both families utilizing dioxygen as electron acceptor [43]. The intradiol dioxygenases, typified by catechol 1,2-dioxygenase, cleave the carbon–carbon bond between the phenolic hydroxyl groups to yield muconic acid as the product [41]. The extradiol dioxygenases, typified by catechol 2,3-dioxygenase, cleave the carbon–carbon bond adjacent to the phenolic hydroxyl groups to yield 2-hydroxymuconaldehyde as the product [42]. In this study, the activities of cellular lysates, catechol 1,2-dioxygenase and catechol 2,3-dioxygenase obtained from *P. fluorescens* were determined (Figure 4). Results showed that the activity of catechol 1,2-dioxygenase was high and varied during degradation process, while the activity of catechol 2,3-dioxygenase was undetected. This indicated that the cleavage of the benzene ring occurred mainly at the *ortho-*position by catechol 1,2-dioxygenase.

Catechol 1,2-dioxygenase activity rose quickly in the first 4 h along with the continuous consumption of phenol (Figure 4). After 4 h, catechol 1,2-dioxygenase activity decreased gradually due to the consumption of phenol. However the co-metabolic degradation of TCE did not cease (Figure 4). Low levels of catechol 1,2-dioxygenase also could assist TCE degradation. This suggests that the presence of phenol is a necessary factor for the generation of catechol 1,2-dioxygenase but is not a necessary factor for TCE continuous degradation during an extended period of time. Actually, TCE can be co-metabolized by resting cells carrying oxygenase without growth substrate. In the study of Kneidel and Yang [44], biodegradation of TCE by resting cells of methanotrophic *Methylosinus trichosporium* OB3b PP358, which constitutively excreted soluble methane monooxygenase (sMMO), was observed.

TCE co-metabolic degradation efficiency was 70.8% after 72 h of degradation. The theoretical value of chlorine ion due to complete dechlorination of the corresponding amount of TCE should be 2.12 mg/L; while the measured value of chlorine ion was 2.30 mg/L. This means that all the chlorine atoms that had been attached to TCE were transformed to chlorine ions. The less chlorinated products, c-DCE, t-DCE, l,l-DCE and VC were measured and could not be detected, suggesting that dechlorination was complete. Gossett found that VC could be degraded aerobically even at very low oxygen concentration [45]. Moreover, total organic carbon (TOC) and pH were measured before and after the experiment, at 0.183 ± 0.006 *vs.* 0.103 ± 0.005 mg/L and 7.01 ± 0.01 *vs.* 6.10 ± 0.02, respectively. These data suggest that some short-chain organic acids may exist as intermediates at the end of experiment.

### 2.4. Ternary and Quaternary Co-Metabolic Degradation Systems of Phenol, TCE, Nutrient Broth and Gasoline

Gasoline is a common pollutant in soil and groundwater, and some constituents of gasoline have been reported to be the growth substrates for TCE co-metabolism [23,30,46,47]. Theoretically, gasoline may act as a growth substrate for TCE degradation. In this study, the TCE co-metabolic degradation in the presence of different concentrations of gasoline was studied (Table 1). In the presence of gasoline, TCE could not be degraded substantially, and a degradation rate of 28% was achieved in the presence of 100 mg/L gasoline. The addition of NB could not enhance the degradation rate of TCE in the presence of gasoline. To improve the degradation of TCE, 100 mg/L of phenol was introduced to the TCE and gasoline system; the degradation rate of TCE was enhanced greatly, and TCE could be degraded by 59%, 45% and 47%, at gasoline initial concentrations of 10, 50 and 100 mg/L, respectively. Simultaneous addition of phenol and NB increased TCE degradation efficiency but not as well as those by the single addition of phenol. On the other hand, satisfying degradation rates of gasoline could always be achieved whether adding phenol or not when gasoline concentration was 10 mg/L (Table 1). However the addition of NB inhibited gasoline degradation. Hence, the mutual effects between different substrates were complicated. In the study of Chang *et al.* [48], two *Pseudomonas* species were isolated from an aerobic pilot-scale fluidized-bed reactor treating groundwater containing benzene, toluene, and *p*-xylene (B, T and X). Batch tests using bi-substrates (B + T, T + X or B + X) revealed that both competitive inhibition and co-metabolic degradation patterns could occur. Shim *et al.* [19] examined the ability of four pure cultures isolated from soils potentially contaminated with gasoline compounds to degrade BTEX (benzene, toluene, ethylbenzene, and three isomers (*ortho*-, *meta*- and *para*-) of xylene), TPH (total petroleum hydrocarbons), and TCE at different concentrations. TPH showed similar degradation rates in the mixtures of TPH + BTEX, TPH + TCE and TPH + BTEX + TCE at different concentration ratios; while BTEX showed significantly greater degradation rates when they co-existed with low levels (<15 mg/L) of TCE as compared with those in the mixture of TPH + BTEX and TPH + BTEX + TCE. The degradation rates of TCE followed the order of BTEX + TCE > TPH + BTEX + TCE > TPH + TCE. It can be concluded that TPH is not a good substrate as BTEX and the mutual influences among different substrates depend on both their degradability and relative concentrations. Similarly, in the present study, gasoline is not a good substrate for TCE co-metabolism, however TCE co-metabolism in the presence of gasoline could be enhanced by the further introduction of phenol. In another study by Tobajas *et al.*, it was found that the addition of glucose could further enhance the co-metabolic degradation of 4-chlorophenol in the presence of phenol [49]. In the present study, the amount of NB was much greater than gasoline, and NB was more easily utilized by microbes than gasoline was; therefore NB was preferentially consumed, and the gasoline remained. These results demonstrate that it is possible to remove combined pollution of TCE and gasoline.

## 3. Experimental Section

### 3.1. Microorganism Culture

In this study, *Pseudomonas fluorescens* was used as the active strain for the aerobic co-metabolic degradation of TCE. The seed culture of *P. fluorescens* used for this study was commercially available (China General Microbiological Culture Collection Center, Beijing, China). The pure culture was obtained as vacuum freeze-dried powder kept in a serum bottle and was kept in the laboratory at 6–10 °C before recovering cultivation. The bacteria powder was recovered according to the guideline of the supplier. Suitable amounts (0.3–0.5 mL) of liquid medium of nutrient broth (see below) were dripped into the serum bottle under sterilized conditions to suspend the powder. The bacteria suspension was shaken gently and transplanted into two medium plates, which were cultivated immediately at 30 °C.

### 3.2. Culture Media

Two kinds of microbial culture media were used. One was mineral salt medium (MSM) [50]. One liter MSM at pH 7.2–7.4 contained: 5 mL phosphate buffer solution (KH_2_PO_4_, 8.5 g/L; K_2_HPO_4_·H_2_O, 21.75 g/L; Na_2_HPO_4_·12H_2_O, 33.4 g/L; NH_4_Cl, 5.0 g/L); 3.0 mL MgSO_4_ solution (22.5 g/L); 1.0 mL FeCl_3_ solution (0.25 g/L); 1.0 mL CaCl_2_ solution (36.4 g/L); 1.0 mL trace element solution (MnSO_4_·H_2_O, 39.9 mg/L; ZnSO_4_·H_2_O, 42.8 mg/L; (NH_4_)_6_Mo_7_O_24_·4H_2_O, 34.7 mg/L). The other was nutrient broth (NB) consisting of 3.0 g beef extract, 10.0 g peptone and 5.0 g NaCl per liter of water. The pH of the medium was adjusted to around 7.0 by adding either HCl or NaOH. All the apparatus and media were autoclaved in advance.

### 3.3. Experimental Procedure

Designated amounts of phenol and TCE were aseptically added to 20 mL of the bacterial suspension in a 40 mL serum bottle. After the serum bottle was covered by screw caps with Teflon-lined silicon septa, it was inverted several times by hand and placed on an orbital shaker operated at 150 r/min and 30 °C. Kinetic experiments lasted for 5 days with TCE concentration determined every day. Other condition optimizing experiments lasted for 3 days based on the result of the kinetic test. The experiment was repeated at different initial concentrations of TCE (0.1–100 mg/L) and phenol (0–500 mg/L), initial organism density (6.3 × 10^5^–1.26 × 10^8^ cell/mL), different nutrient conditions (addition of NB or not), pH (4–9), and salinity (0.045%–3%) to optimize the degradation of TCE. After preliminary experiments, the entire optimizing experiments were repeated by changing one specific parameter and setting other parameters at their optimal values acquired in the preliminary experiment. To check the potential of gasoline as a growth substrate, different concentrations (10–100 mg/L) of gasoline was introduced instead of phenol. Moreover, phenol and NB were added into the system of TCE and gasoline to improve the co-removal of TCE and gasoline. Each experiment was conducted in triplicate.

### 3.4. Analytical Method

After the experiment, 20 mL of *n*-hexane was injected into the serum bottle through the Teflon-lined silicon septa by syringe to extract TCE and gasoline (in some experiments). The extract was assayed by gas chromatography for TCE and by UV spectrometer for gasoline. Using this method, more than 93% TCE and more than 92% gasoline could be recovered.

The concentrations of TCE and its possible less chlorinated intermediates (c-DCE, t-DCE, l,l-DCE and VC) were measured using a gas chromatograph (Agilent 7890A, Agilent Technologies Corporation, Wilmington, DE, USA) equipped with an electron capture detector (μ-ECD) and a capillary column (HP-5; 30 m × 0.320 mm I.D. with a stationary-phase film thickness of 0.25 μm). One microliter of liquid sample was injected by an autosampler injector equipped with a tapered microsyringe (Agilent Technologies Corporation, Wilmington, DE, USA). The concentration of gasoline was measured using a spectrophotometer (T6 New Century, Beijing Purkinje General Instrument Corporation, Beijing, China) at 225 nm.

To avoid the loss due to TCE evaporation, a closed system was used. At the end of the experiment, oxygen was measured (HQ30d, Hach Corporation, Loveland, CO, USA). The oxygen was not less than 3.63 mg/L, which ensured the aerobic condition of the reaction system, unless the lower value was pointed out in certain experiments. The magnitude of OD was measured at 600 nm using a spectrophotometer to quantify the microorganism density, and the relative standard deviation of this method is 30%. The concentration of chlorine ion generated by dechlorination of TCE was measured by an ion chromatograph (DX-120, Dionex Corporation, Sunnyvale, CA, USA). Total organic carbon (TOC) was measured using a TOC analyzer (Multi N/C 3100; Analytic Jena, Jena, Germany) to evaluate the extent of oxidation.

### 3.5. Assay of Enzyme Activities

Bacterial cells were harvested by centrifugation at 5000× *g*, washed twice with 20 mM phosphate buffer (pH 7.0), and resuspended in the same buffer. This suspension was used to assay catechol 1,2-dioxygenase and catechol 2,3-dioxygenase. Cells were disrupted using a sonifier (Branson Ultrasonics Corporation, Danbury, CT, USA). The cellular lysates were centrifuged at 19,000× *g* for 20 min, and the supernatant was used for enzyme assays. The reaction mixture (total 3.0 mL) contained 2.0 mL phosphate buffer, 0.6 mL 1 mM catechol, 0.2 mL deionized water and 0.2 mL cellular lysates. The reaction was allowed to proceed at 22 °C for 1 min. Catechol 1,2-dioxygenase and catechol 2,3-dioxygenase activities were determined by measuring the production of muconic acid at 260 nm and 2-hydroxymuconic semialdehyde at 375 nm, respectively [51]. Protein concentration was determined according to Bradford method [52].

## 4. Conclusions

This study investigated the aerobic co-metabolic degradation of TCE in aqueous phase by *Pseudomonas fluorescens* using phenol as the main growth substrate. TCE co-metabolic degradation rates reached maximum when the initial OD_600_ of the culture was 0.14, phenol concentration 100 mg/L, and TCE initial concentration 0.1–10 mg/L. TCE degradation efficiency was satisfactory within a wide acid-base range (pH = 5–9) and salinity (0.045%–3%) range, which indicates its applicability in salty-alkaline seashore areas. The addition of nutrient broth did not enhance TCE degradation singly, but promoted TCE co-metabolic degradation using phenol as growth substrate. The primary cause of the nutrient broth promotion effect was the increase of cells, which generated an active enzyme system stimulated by phenol. Correspondingly, the addition of nutrient broth improved transformation yield but inhibited transformation capacity. Catechol 1,2-dioxygenase was the key enzyme in TCE co-metabolic degradation. The presence of phenol was a necessary factor for the generation of catechol 1,2-dioxygenase; however, catechol 1,2-dioxygenase co-metabolism activity can be maintained for a certain time without phenol. The dechlorination of TCE was complete, and all the chlorine atoms, which had existed in degraded TCE were transformed to chlorine ions. The co-metabolic degradation of TCE was slow in the presence of gasoline, while the addition of phenol led to an enhanced removal of both TCE and gasoline. These results provide a promising technology for the simultaneous removal of combined pollution of TCE and gasoline *in-situ* or *ex-situ*.

## Figures and Tables

**Table 1.** Degradation rates (%) of TCE and gasoline using *P. fluorescens* in different systems with varied substrates and gasoline concentration.

Substrates	G. 10 mg/L		G. 50 mg/L		G. 100 mg/L	
		
	TCE	G.	TCE	G.	TCE	G.
TCE + G.	23.9 ± 0.8	81.3 ± 1.6	24.9 ± 0.5	28.5 ± 0.8	28.5 ± 0.4	20.7 ± 0.4

TCE + G. + P.	58.8 ± 1.1	68.8 ± 1.2	44.9 ± 0.8	18.0 ± 0.3	47.0 ± 0.8	21.6 ± 0.5

TCE + G. + NB	23.7 ± 0.5	31.3 ± 0.9	25.1 ± 0.5	21.1 ± 0.5	25.1 ± 0.6	15.2 ± 0.5

TCE + G. + P. + NB	45.0 ± 0.9	33.3 ± 1.1	44.5 ± 0.9	8.8 ± 0.1	32.5 ± 0.9	5.5 ± 0.1

G., P., and NB mean gasoline, phenol and nutrient broth, respectively.
